# Unusual intravesical foreign bodies: a report of two cases

**DOI:** 10.11604/pamj.2023.45.148.39227

**Published:** 2023-08-03

**Authors:** Abdoul-Karim Pare, Adama Ouattara, Delphine Ye, Abdoul Kader Tapsoba, Hilaire Sawadogo, Sinaly Soare, Mickael Rouamba, Babagana Mustapha Abubakar, Mohamed Simpore

**Affiliations:** 1 Division of Urology, Souro Sanou University Teaching Hospital, Bobo-Dioulasso, Burkina Faso,; 2 Division of Surgery, Regional Hospital, Dori, Burkina Faso,; 3 Division of Surgery, Federal Medical Center, PMB 02, Nguru, Yobe State, Nigeria

**Keywords:** Foreign body, urethra, endoscopic extraction, case report

## Abstract

The introduction of a foreign body into the urethra is an exceptionally rare occurrence. It is often secondary, either to erotic curiosity or to psychiatric disorders. The symptomatology is multiple and the diagnosis is aided by radiologic imaging. The extraction is most often done by endoscopic. The management of these patients must be done systematically and will need psychiatric assessment. We report the case of two patients who inserted a foreign body into their urinary bladder. One was 22-year-old and the second was 20-year-old and all with no history of psychiatric disorders. The first accidentally inserted a pencil into the urethra while trying to flatten a vulvar papule. The second inserted a piece of broom through playful games. The foreign bodies were extracted endoscopically in both cases under rachianesthesia. The postoperative course was uneventful and both patients were referred to a psychiatric clinic for evaluation after discharge.

## Introduction

The introduction of a foreign body in the urinary bladder by the urethra is an exceptionally rare occurrence. It may occur by self-insertion [1]. Most cases occur in a psychiatric patient or during erotic stimulation or other forms of sexual abuse [2]. The presentation may be myriads. However, the diagnosis can be made by radiologic imaging. The hallmark of the management is an endoscopic evaluation that can serve both diagnostic and therapeutic purposes [3]. Management requires a detailed history taking as well as psychiatric evaluation and thorough examination [2]. Few reported cases of the introduction of a foreign body into the urinary bladder exist in the literature [2,4]. Here, we report two cases of intravesical foreign bodies.

## Patient and observation

### Patients' information

**Case 1:** the first patient was a 22-year-old female, student, who has no significant past medical history presenting with a 3-day history of foreign body insertion into the bladder through the urethra. The patient claims that the pencil accidentally went into the external urethral meatus when she was using it to scratch around her vulva region to relieve some itching as a result of vulva rashes. She complained of mild suprapubic pain and burning micturition. There was no significant psycho-social history.

**Case 2:** the second patient was a 20-year-old female, a student who presented to the emergency room with a 1-day history of the introduction of a piece of a broom into the bladder through the urethral meatus. She had no history of psychiatric disorders. She alleged accidentally introduced the piece of broom for sexual gratification

### Clinical findings

**Case 1:** physical examination was normal apart from moderate hypogastric tenderness. Examination of the urethra and external genitalia was normal.

**Case 2:** the second patient's clinical examination was unremarkable. The gynecological examination was normal.

**Diagnosis assessment:** laboratory investigations found normal values for serum creatinine, hemoglobin, and white blood cells, urine analysis, and urine culture in both cases.

**Figure 1:**
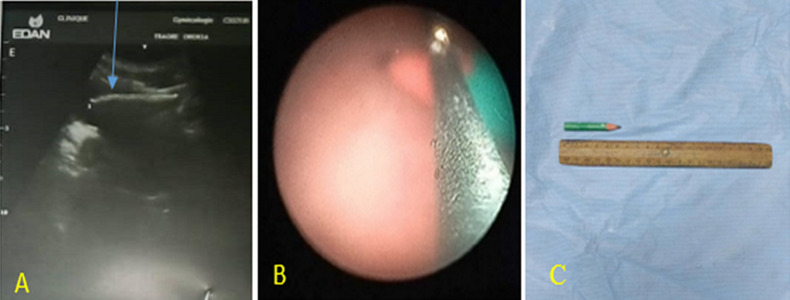
diagnosis and management of intravesical pencil; A) arrow showing foreign body on pelvic ultrasound; B) endoscopic view of the pencil with tripod gripper; C) the removed pencil measuring approximately 50 mm

**Case 1:** the pelvic ultrasound showed a hyperechoic image 49 mm long hanging from the bladder dome (Figure 1 A). The diagnosis of intravesical foreign body was made. Cystoscopy was performed under local anesthesia and antibiotic prophylaxis. Urethrocystoscopy showed a greenish-colored pencil (Figure 1 B) and was removed ( Figure 1 C).

**Figure 2:**
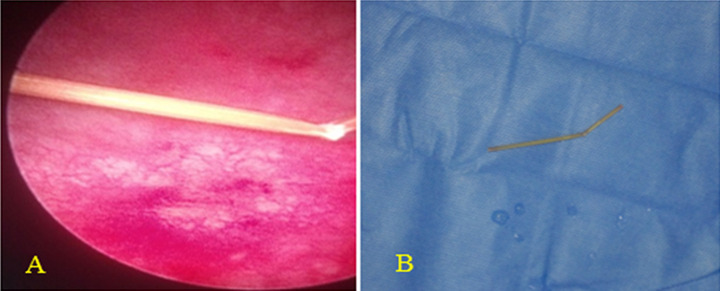
endoscopic diagnosis and removal of intravesical piece of broom; A) endoscopic view of a piece of broom; B) the removed piece of broom measuring approximately 60 mm

**Case 2:** the pelvic ultrasound was not done and urethrocystoscopy was performed under local anesthesia for diagnosis and the foreign body was identified (Figure 2 A). It is a piece of broom.

### Therapeutic interventions

**Case 1:** urethrocystoscopy was done under local anesthesia, and the bladder mucosa was normal. The pencil was grasped with forceps. The foreign body was successfully removed without any difficulty through the cystoscope. Pencil measuring approximately 50 mm.

**Case 2:** urethrocystoscopy was done under local anesthesia, the foreign body was grasped with forceps and extracted without difficulty (Figure 2 B).

**Follow-up and outcomes:** the postoperative course was uneventful. They were discharged from the hospital on the first postoperative day and were referred to the psychiatrist for further evaluation and psychological support. At 3 months of follow-ups, they did not have any complaints.

**Patient perspective:** the patients were happy with the successful outcome of the endoscopy.

**Informed consent:** written informed consent was obtained from the patients for participation in our study.

## Discussion

The self-introduction of a foreign body into the urinary bladder is rare. The actual incidence is difficult to specify with precision [5,6]. In most circumstances, there was some background psychiatric disorder such as mental retardation, dementia, schizophrenia, or major depression. Some incidences occurred for sexual gratification [7]. In the two cases we presented, the most likely reason for the insertion of the foreign body into the urinary bladder was psychiatric disorder and sexual gratification in the 1^st^ and 2^nd^ cases respectively. However, cases of intrauterine device migration have been described by Kambou *et al*. in Burkina [5], Ndoye *et al*. in Senegal [8], and Dahami *et al*. in Morocco [9]. The nature of the objects varied, there were reported cases of various objects in the urinary bladder such as a hairpin, a condom, a fishing line, a nail, a thermometer, a pen or a pencil as in our 1st case, and also a piece of a broom as in our second case [10,11].

Usually, the patients present with dysuria, perineal pain, urethral discharge, or even hematuria as in our second case [11]. The diagnosis is usually confirmed by radiologic imaging (plain Abdominal X-ray, abdominal and pelvic ultrasound, or occasionally even computed tomography) [1]. Ultrasonography is usually able to localize the foreign body to the urinary bladder and determine the exact size and number but is unable to evaluate the exact nature [4]. In our first case, the diagnosis was confirmed by pelvic ultrasound which showed a hyperechoic intravesical foreign body that was 52 mm in length. Ultrasonography was not done in the second patient.

Cystoscopy has both diagnostic and therapeutic roles, it can also show any urinary bladder complication or pathology [12]. In the two cases we presented, cystoscopy helps to confirm the diagnosis, assess the urinary bladder, and also extract the foreign body. Endoscopic foreign body extraction remains the standard minimally invasive treatment [13]. However, open surgery may be an alternative option in some cases due to the nature or volume of the foreign body or because of the unavailability of endoscopic equipment [7]. Several complications may follow the presence of foreign bodies in the urinary bladder. Such as urinary tract infection, stone formation, eventual erosion into the bladder lumen, and acute urinary retention [11]. In our study only a second patient presented hematuria. However, urine microscopy and culture were negative for the infection for the two patients. The psychiatric evaluation of these patients is very important so as not to miss any serious psychiatric disorder in the patient and also to avoid any medicolegal issues in the management of these patients [14]. In the two cases we presented, we referred both to psychiatry for further evaluation.

## Conclusion

Self-introduction of intravesical foreign bodies through the urethra is usually done for sexual gratification, however, it has been reported in patients with background psychiatric disorders. Diagnosis is usually confirmed by radiologic evaluation. Urethrocystoscopy plays both diagnostic and therapeutic roles. All patients should be referred to psychiatrists for further evaluation.
